# Planning for Implementation Success of an Electronic Cross-Facility Health Record for Pediatric Palliative Care Using the Consolidated Framework for Implementation Research (CFIR)

**DOI:** 10.3390/ijerph19010453

**Published:** 2022-01-01

**Authors:** Theresa Sophie Busse, Sven Kernebeck, Larissa Alice Dreier, Dorothee Meyer, Daniel Zenz, Peter Haas, Boris Zernikow, Jan Peter Ehlers

**Affiliations:** 1 Department of Didactics and Educational Research in Health Science, Faculty of Health, School of Medicine, Witten/Herdecke University, 58448 Witten, Germany; sven.kernebeck@uni-wh.de (S.K.); jan.ehlers@uni-wh.de (J.P.E.); 2 PedScience Research Institute, 45711 Datteln, Germany; l.dreier@pedscience.de (L.A.D.); d.meyer@pedscience.de (D.M.); b.zernikow@kinderklinik-datteln.de (B.Z.); 3 Department of Children’s Pain Therapy and Pediatric Palliative Care, Faculty of Health, Witten/Herdecke University, 58448 Witten, Germany; 4 Smart-Q Softwaresystems GmbH, 44801 Bochum, Germany; zenz@smart-q.de; 5 Department of Medical Informatics, Dortmund University of Applied Sciences and Arts, 44139 Dortmund, Germany; haas@fh-dortmund.de; 6 Pediatric Palliative Care Centre, Children’s and Adolescents’ Hospital, 45711 Datteln, Germany

**Keywords:** digital health, health information technology, design thinking, consolidated framework for implementation research, implementation, palliative care, palliative medicine, pediatrics, electronic health record

## Abstract

Pediatric palliative care (PPC) patients require years of care across professions and sectors. Sharing treatment-related information and communicating among different PPC professionals is critical to ensure good quality of care. In Germany, this communication is mostly paper-based and prone to errors. Therefore, an electronic cross-facility health record (ECHR) was participatorily designed with users, wherein information can be shared and PPC professionals can communicate with each other. As this form of electronic health record differs from existing models in Germany, there is a need for successful implementation to ensure a positive impact. Therefore, the facilitators and barriers to the implementation of ECHR in PPC were examined. Using the consolidated framework for implementation research (CFIR), transcripts of 32 interviews, 3 focus groups, and 20 think-aloud studies with PPC professionals were analyzed. CFIR indicated that the ECHR-design was viewed positively by users and can be a facilitator for implementation. Barriers exist, mainly due to the fact that the implementation is not planned, the use of the ECHR involves effort, costs are not covered, and all users must be motivated to use the ECHR for functionality. CFIR helps uncover the crux of the issues that need to be considered when planning ECHR implementation to improve care in PPC.

## 1. Introduction

Pediatric palliative care (PPC) follows a holistic approach to care for children, young adults, and adolescents with life-limiting or life-shortening illnesses [1]. Care is provided for many years [2] and in sequences of treatment episodes from various inpatient and outpatient providers, such as pediatricians and general practitioners from medical offices (MO) and general outpatient PPC teams at home [3]. The general conditions of PPC patients and their relatives are variable. Support from specialist physicians, therapists, outpatient hospice services, and specialized outpatient PPC (SOPPC) teams is often necessary. It is also possible to be admired in a pediatric hospice to relieve the relatives and support them. In case of the intensification of symptoms, PPC patients can be admitted to a PPC unit (PPCU) [2]. The continuity of care can be seen as challenging when PPC patients change across PPC settings [4].

To support the exchange of information between PPC professionals, a shared electronic cross-facility health record (ECHR) was designed in a participatory design approach with future users [5,6]. The resulting ECHR differs from currently existing ECHRs in Germany that pursue a similar purpose: existing ECHRs share information regarding an indication-specific and time-limited treatment episode as a case (e.g., hospital stay from 1 January 2021 to 1 August 2021) with post ambulatory treatment for some weeks. After the end of treatment and support of information transmissions of the case-related information between health care professionals, during the treatment episode, the case in the ECHR is closed [7]. In contrast, the developed ECHR understands the life-limiting or life-shortening illness as a case, and information can be shared continuously until the end of the life of a PPC patient. Information that has already been entered remains visible and can be supplemented with additional, more recent information. Likewise, longitudinal views and evaluations of certain aspects are possible.

Implementing such an ECHR would offer PPC professionals the opportunity to share information promptly between different settings and health care professionals. This could lead to a more holistic overview of PPC patients and improved communication between PPC professionals while reducing the duplication of work. A systematic review on implementing documentation systems in health care facilities showed 57 different barriers. The most common ones were user resistance, lack of skills or concern for return on investment, and a lack of administrative and policy support [8]. From the perspective of nurses, training with the software, adapting the software to the workflow, and adapting the internal organizational structure to the software could impact the success of the implementation [9]. Another systematic review of the factors influencing the implementation of electronic health records showed that technology, external setting, internal setting, users of the technology, and the implementation process are crucial for the success of the implementation [10].

Therefore, it is necessary to identify facilitators and barriers in the implementation process and to be able to develop a context-specific implementation strategy. It can be assumed that facilitators and barriers influencing the implementation process can be transferred, as with electronic health records. However, the use of such an ECHR has not yet occurred in Germany in the sense of PPC. Preparing a strategic plan in the pre-implementation phase of an electronic health record is essential [11].

A theoretical foundation is useful to determine the extent to which the implementation of the new ECHR is possible. A frequently used framework to assess facilitators and barriers before implementation and thus create incentives to improve them is the consolidated framework for implementation research (CFIR) [12]. CFIR is often used in digital health to prepare the planning of implementation and draw attention to the critical points in the process [13].

This article aims to use the CFIR to identify the facilitators and barriers to ECHR implementation for PPC in advance and as a basis for implementation planning. This involves implementing the health care professionals of one PPCU, three SOPPC teams, and the corresponding physicians in MO in a region in Germany. The facilitators and barriers are reported based on the qualitative data collections carried out in the participatory design process of the ECHR. This article’s findings will examine which areas still need action for successful implementation and which areas have strengths that can support implementation.

## 2. Materials and Methods

This study follows the development of an ECHR in the ELSA-PP (Acronym for the German term of “ECHR for PPC”) project to critically identify facilitators and barriers to ECHR implementation in advance of implementation planning. Ethical approval was obtained from the Ethics Committee of Witten/Herdecke University in Germany (approval code: 35/2019).

### 2.1. ECHR

**Figure 1:**
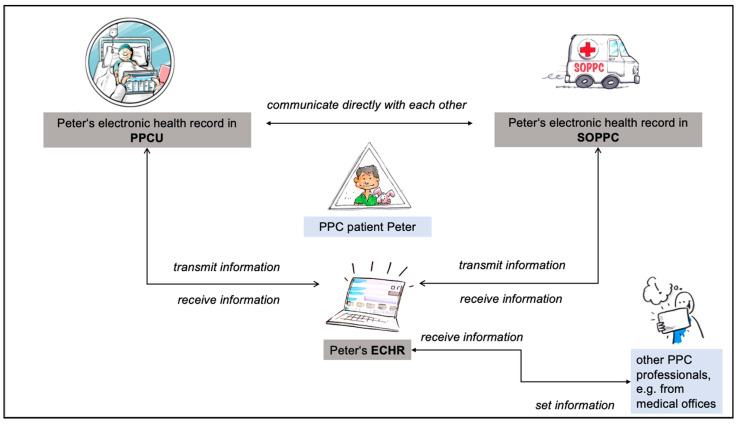
Data exchange between PPCU, SOPPC, and ECHR (drawings by Dagmar Gosejacob and Ralf Marczinczik for the project).

Web-based ECHR serves PPC professionals to read patient-related information and to share it between different settings and stakeholders. The ECHR consists of an overview of all contacts between health care professionals (among themselves) as well as with the patients and their relatives, information on diagnoses, medication, prescriptions of medical aids, and a calendar. Additionally, it includes a symptom-related treatment plan, information on specialized care (wound documentation, ostomy, ventilation machines), and an overview of persons with access authorization. In the ECHR, it is also possible to exchange messages with other PPC professionals in the system and to take notes. Information from the electronic health records of a PPCU and SOPPC are synchronized bidirectionally. For example, if it is entered in the electronic health record on the PPCU that a drug has been restarted, this information will also appear in the ECHR. If it is documented in the ECHR that the child has been given a new diagnosis, this will also appear in the documentation of the PPCU and SOPPC. Other PPC professionals can then supplement the comprehensive information displayed in the ECHR via the ECHR (Figure 1).

The ECHR should thus enable more information to be obtained, duplicate examinations, conversations to be avoided, and treatment to be planned together. The aim is to have one place where all information is combined from different settings. For example, to have the most up-to-date medication plan with all tracked changes available for everyone involved.

There is no legal requirement to document in the ECHR, although improving care through its use is likely. However, in a PPCU, none of the employees refused to use an electronic health record. Here, the success and usefulness of the ECHR depends on the information that is entered into the system. It is therefore crucial to plan the implementation to ensure that the system is well accepted.

### 2.2. Theoretical Framework

The CFIR was selected to offer an overview in which area adjustments/planning are necessary for implementation. It comprises 39 constructs regarding the influence on implementation and effectiveness in five major domains (Intervention Characteristics, Outer Setting, Inner Setting, Characteristics of Individuals, Process) [12]. Using a snowball approach, the authors of CFIR identified 19 published theories regarding implementation theory development and combined the respective constructs into the CFIR. Some constructs appeared in many of the studies, while others were rarely represented [12].

Prior to implementing the ECHR, the CFIR will be used as a guide for analyzing facilitators and barriers for implementation as recommended [12]. A systematic review showed that this is the most common form of CFIR use [14].

The respective constructs of the CFIR designate an institution as the entity that will perform an implementation. While multiple institutions and individuals will use ECHRs, the definition of institutions has been reworded. The “institution” hereafter means all PPC professionals who jointly care for a patient and his:her relatives.

### 2.3. Participants

In the sense of participatory design, potential future users (nurses, physicians, secretaries, and psychosocial staff) were involved in developing the ECHR [5,6]. This included PPC professionals from one PPCU and three SOPPC teams, and physicians from MOs (pediatrists and general practitioners) with experience with PPC patients. In addition, PPC professionals who worked on both the PPCU and one of the SOPPC teams were asked to participate. The SOPPC teams involved were located in neighboring cities to the PPCU and worked closely with them. Physicians from MOs also provided regular care for PPC patients together with the PPCU.

### 2.4. Data Collection

The basis for identifying facilitators and barriers to implementing the ECHR is the data from the previously conducted participatory development of the ECHR, described in more detail in the associated publications [5,6]. This was carried out based on adapted design thinking (DT) [15]. DT is an iterative process focusing on understanding specific problems and the development of effective solutions [16]. The DT model followed to develop the ECHR combined the needs assessment, conceptualization, and iterative improvement using interviews, focus group interviews, and think-aloud sessions (TA). To identify facilitators and barriers to the implementation of the ECHR, all transcripts were analyzed again and assigned to the constructs of the CFIR.

### 2.5. Data Analysis

Interviews were conducted by researchers with experience in qualitative research with 32 PPC professionals, 3 focus groups with 13 users, and think-aloud studies with 20 PPC professionals. This included a total of three individuals from administrative staff, 26 nurses, 5 psychosocial workers, and 27 medical professionals in all data collection steps. Some individuals participated in more than one data collection. Thirty-five of the participating individuals worked at the PPCU, thirteen in SOPPC teams, and thirteen in MOs. Further information on the characteristics of the users can be found in the corresponding papers [5,6]. The transcripts of the interviews, focus groups, and TAs with PPC professionals were analyzed using MAXQDA software (VERBI Software GmbH, Berlin, Germany) [17].

The analysis was performed deductive by T.S.B. and S.K., using the constructs of the CFIR as categories [18]. This included all statements about the construct that could be seen as barriers and statements that can be seen as facilitators for the implementation (see Appendix A).

The original quotes were translated into English during the preparation of the manuscript. The users were assigned pseudonyms following the structure “data collection method_profession_setting_interview number” (e.g., TA_nurse_PPCU_03). The respective data collection methods were abbreviated here: think-aloud session as TA, interview as I. The particular settings are subdivided into users from the PPCU (PPCU), SOPPC teams (SOPPC), and medical offices (MO).

## 3. Results

A total of 848 codes were assigned in a deductive approach, using the CFIR constructs as main and subcategories (range: 1 to 42 codes per interview/TA). No codes were assigned to the CFIR constructs Aaptability, Structural Characteristics, Readiness for Implementation, Individual Identification with the Organization, Planning, Engaging, Executing, and Reflecting or Evaluating in the transcripts.

**Figure 2:**
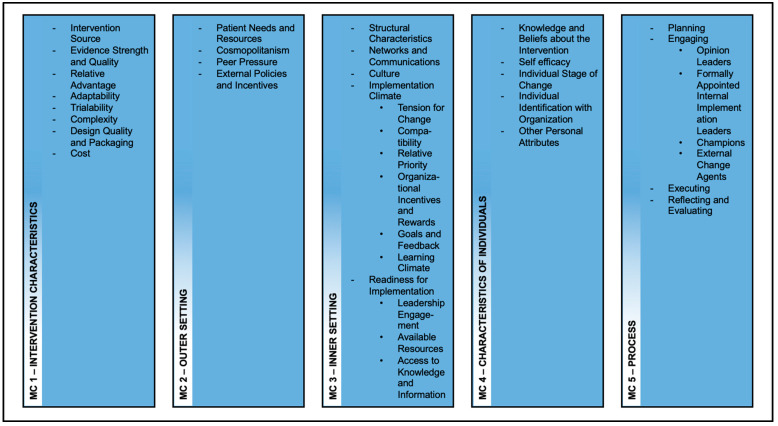
Codesystem from MAXQDA—oriented to CFIR.

The presentation of results is based on the main categories (MC), which are further split into subcategories (SC) (Figure 2), based on CFIR and adapted to the setting.

### 3.1. MC1—Characteristics of the Intervention

MC 1 is devoted to how the intervention is equipped in terms of participation, quality, and validity, whether it has advantages over alternative solutions, and to what extent it can be adapted in the future. In addition, the question concerning whether the ECHR could already be tested and whether the complexity, design, and price are appropriate is also examined.

#### 3.1.1. SC 1.1—Intervention Source

This includes information about the perception of users whether the ECHR is ex- or internally developed. As described above, users were involved in the development process. Users indicated in the interviews that they felt it was positive to be involved in the process.

*I think that’s very good because I don’t do it for myself alone. I can talk to you directly and ask again, and you get my thoughts directly.*

(TA_nurse_PPCU_06)

#### 3.1.2. SC 1.2—Evidence Strength and Quality

The SC comprises users’ perceptions of the quality and validity of evidence supporting the belief that the ECHR will have desired outcomes.

Users felt that the ECHR provided structured treatment documentation and minimized effort through automatic transfer in the TA. They also indicated that ECHR improves the safety of care, reduces redundancy, and allows for a better overview of the patient. Additionally, they said that a quality feature is transferring information in compliance with legal data protection regulations.

#### 3.1.3. SC 1.3—Relative Advantage

A relative advantage describes the advantage of implementing ECHR versus an alternative solution.

Users described the current information exchange as problematic due to the fact that there is sometimes a lack of information (e.g., contact person, information already exchanged, duplicated diagnoses). Data overload can lead to loss of important information, and the time required to exchange information is high. The advantages of ECHR are that it is appropriate for the complexity of PPC patients and that it is designed to be very clear and cross-system. Another advantage was that the ECHR is designed to extend beyond individual hospital stays. Existing ECHRs that users were aware of were always tied to a hospital stay and did not continue after that.

*We also have an ECHR version in [name of city]. That’s not so complex. It’s more for acute patients and so on. I think because they are very special patients, with whom there are also more than… Um, if I compare it with [name of the city], it is also sometimes used for very sick children, but there is no SOPPC team attached to it or something. So, I think this version for SOPPC teams is very good in terms of complexity and also well-structured, so you actually find what you need.*

(TA_physician_MO_16)

The users feared that errors could occur if too many people had access to the ECHR. Additionally, they cited the difficulty of making the personal transition from analog to digital.

#### 3.1.4. SC 1.4—Trialability

The ability to test the ECHR on a small scale in the organization and to be able to reverse course (undo implementation) if warranted is named trialability in the CFIR.

The users rated it positively to be able to test the ECHR in advance as part of the DT process.

*I thought it was good that I could try it out a little bit. You just click on it and see what happens. And then I get a few explanations about what’s behind it or what could be behind it, which I found good. I liked that very much. I was happy to invest that time there.*

(TA_physician_MO_17)

In addition, it was mentioned that the assessment of content in the TA is sometimes difficult. The users here stated that they received many different new impressions within the framework of the TA. In this area of participatory development, a comprehensive content evaluation is only possible to a limited extent due to the limited use case with a persona and the special situation.

#### 3.1.5. SC 1.5—Complexity

This SC includes statements about the perceived difficulty of the ECHR, reflected by duration, scope, radicalness, disruptiveness, centrality, intricacy, and number of steps required to implement.

Many users saw a necessity for introduction and further training in the system due to the high complexity of the ECHR. The users noted that the effort required to use the system would be high. It was also mentioned that it is positive that the ECHR is designed in a complex way. In this way, it could sufficiently reflect the complexity of the PPC. However, users also indicated that one person must manage the ECHR to avoid duplication of content and maintain structures.

#### 3.1.6. SC 1.6—Design Quality and Packaging

Design quality and packaging describe the perceived excellence in how the ECHR is bundled, presented, and assembled.

The PPCU and SOPPC users felt that ECHR was intuitive since it was modeled on existing systems. Users particularly valued the cross-system design as a quality feature of the ECHR.

#### 3.1.7. SC 1.7—Costs

This SC focuses on the costs of the ECHR and costs associated with implementing the ECHR, including investment, supply, and opportunity costs.

Users were concerned that some institutions might not cover costs.

### 3.2. MC 2—Outer Setting

MC 2 collects assessments of how the intervention addresses patient needs, how the organization is networking, what other similar developments already exist that could impact implementation, and how the organization can disseminate interventions in the community.

#### 3.2.1. SC 2.1—Patient Needs and Resources

Based on CFIR, this SC includes statements about the extent to which the needs of patients and their relatives, as well as barriers and facilitators to meet those needs, are accurately known and prioritized by the organization.

Users mentioned the availability of all information to all stakeholders, reduced burden on parents by eliminating frequent medical histories, and collaborated on content across sectors as benefits to patients and their relatives.

*You already know them* [the patients]. *a bit and don’t have to open up a huge anamnesis with the parents and not ask them any questions, but you can simply ask a few things that are already there. You just have to ask: Has anything changed? And then it’s also good because sometimes I find it just annoying for parents who come with a sick child when someone constantly comes in and asks again. How is it and how does it work with the bowel movement, and how does it work here and there? Sometimes you think to yourself: What must they think now? In any case, I think it’s very, very great.*

(I_physician_MO_08)

#### 3.2.2. SC 2.2—Cosmopolitanism

Statements about the degree to which the organization is networked with other external organizations are decisive in this SC.

The organization’s networking with other external organizations is high based on the interviews. Thus, 22 different institutions, individuals, and groups were named with whom a regular exchange takes place. The overview of the respective responsible persons per patient in the ECHR was therefore rated very positively.

#### 3.2.3. SC 2.3—Peer Pressure

This SC is about the mimetic or competitive pressure to implement an ECHR, which is typical since most other key peer or competing organizations have already implemented or are bidding for a competitive edge.

The users mentioned that there is already an ECHR in place used by other organizations, but this does not meet the needs of the PPC. They stated that if different areas develop different ECHR systems, ultimately, no system will prevail. However, ECHR has the advantage that the “case” for which it is designed is the underlying disease.

*Most ECHRs, as far as I know, are created and exist only for a certain time. And then it’s gone again. And that shouldn’t be the case with a chronically ill patient or a palliative patient.*

(I_physician_MO_05)

#### 3.2.4. SC 2.4—External Policy and Incentives

This SC includes external strategies for disseminating the ECHR, including policies and regulations (governmental or other central entity), external mandates, recommendations and guidelines, performance-based pay, collaboratives, and public or benchmark reporting.

The consideration of data protection regulations and data security are the most important aspects from the users’ point of view. They mentioned that it might be necessary to obtain the parents’ consent to use the ECHR and transmit its data. However, there is a great deal of uncertainty among users regarding the extent to which the exchange of data with additional persons complies with data protection requirements.

### 3.3. MC 3—Inner Setting

MC 3 addresses structure, communication, culture, and capacity for change in implementation within the organization. It also looks at how intolerable the current situation is and how high a priority implementation is. Another focus is on assessing the extent to which the ECHR is in line with the individual needs of the organization’s members. The involvement of leaders in the implementation, the resources available for it, and the knowledge provided within the organization are also considered.

#### 3.3.1. SC 3.1—Networks and Communications

This SC focuses on the nature and quality of social networks and (in-)formal communications within the organization.

Communication takes place for many different reasons, such as referral to specialists, clarification of issues, admission to a facility, and more and is time consuming. Communication between organizations is carried out with mail, letters, fax, phone, and face-to-face meetings. However, this is sometimes characterized by a lack of contact persons, unclear responsibilities, and limited accessibility.

*People just call spontaneously, whatever number they find at the moment. And then you think, (sighs) are we responsible for this or is the SOPPC team? So, it’s always a bit difficult to find out who is ultimately responsible for something.*

(I_nurse_PPCU_03)

#### 3.3.2. SC 3.2—Culture

Statements about the norms, values, and basic assumptions of the particular organization are the focus here.

Users emphasized that the need for personal contact to share patient information remains even with the use of ECHR and cannot be completely replaced by technology.

#### 3.3.3. SC 3.3—Implementation Climate

This SC includes the CFIR constructs Tension for Change, Compatibility, Relative Priority, Organizational Incentives and Rewards, Goals and Feedback, and Learning Climate. It includes information about the existing climate for the implementation of the ECHR.

Regarding this, users reported that the affinity for technology among users is diverse, which will impact the implementation. The tension for change is high since most users rated the current situation as time-consuming and unsatisfactory.

*It doesn’t work well between the different settings, as it is right now. There are always many problems that one or the other plan is not up to date or missing.*

(TA_nurse_SOPPC_12)

A possible barrier could be that it was suspected that the use of the ECHR would not be equally intensive for all users. In addition, it was suspected that some components would be used only selectively, especially by family doctors, although the ECHR offers a good overview.

Users saw a risk of large amounts of data being transferred from inpatient and outpatient documentation, leading to clutter in the ECHR. The users saw the importance of the implementation within the organization and stated that everyone must work with the system to keep the content current and the ECHR usable.

### 3.4. MC 4—Characteristics of Individuals

MC 4 includes a focus on users’ individual assessment of the ECHR, their self-efficacy, and the personal stage users are in regarding ECHR implementation, as well as other personal attributes and connections to the organization.

#### 3.4.1. SC 4.1—Knowledge and Beliefs about the Intervention

This SC summarizes users’ attitudes toward and value of the ECHR as well as familiarity with facts, truths, and principles related to the ECHR.

Users indicated that the prototype would make it easier for them to evaluate the practicality of the ECHR.

*I think it’s great that I can try out everything and do it. I can’t really say in theory whether something is good or bad. But when there’s a prototype, I can say I’m still missing this and this and this.*

(TA_physician_PPCU_13)

#### 3.4.2. SC 4.2—Self-Efficacy

The individual belief in one’s own capabilities to achieve implementation goals is collected in this SC.

Implementation is made more difficult since time is scarce in everyday work, and using the ECHR system can save time but initially involves a great deal of effort (depending on the affinity for technology). Here, the users indicated that they might find it difficult to use the ECHR under the given circumstances, although they are convinced that it could be helpful.

#### 3.4.3. SC 4.3—Individual Stage of Change

The individual stage of change focuses on the characterization of the phase in which persons use the intervention skillfully, enthusiastically, and sustainably.

The statements on this were quite diverse. Some people described the ECHR system as intuitive to use and the application as not complicated. Others named that they had a better overview in paper documentation, and one has to get used to the system.

#### 3.4.4. SC 4.4—Other Personal Attributes

This SC includes other personal characteristics, such as ambiguity tolerance, intellectual ability, motivation, values, competence, capacity, and learning style.

It was hypothesized that older individuals would be less likely to accept ECHR than younger individuals would. In addition, professionals from the PPCU and SOPPC teams suggested that the system was too complex and time-consuming for family doctors to use.

## 4. Discussion

In this article, a systematic process was described to identify the facilitators and barriers based on the CFIR to the implementation of an ECHR for PPC before implementation planning. In this analysis, factors that could positively or negatively influence the implementation were identified.

The ECHR represents an improvement on the current situation from the perspective of the users, but there are four major challenges regarding the implementation of the ECHR:

First, it could be assumed that there is a need for training on use. This should urgently be considered, as a systematic review identified lack of education and training as the main barrier to implementation. Additionally, a lack of technical expertise and computer skills were found to be common barriers [8]. This should be taken into account when designing the training. In addition to training, knowledge is also a crucial factor. The issue of data protection played an important role for the users. There is already discussion regarding the particular legal and security challenges of shared health records [19]. The focal points seen here are the allocation of responsibility, documentation routines, and integrated or federated access control [20]. These problems coincide with the statements of the users. Although the ECHR has a data protection concept and the applicable regulations in Germany are taken into account, it is interesting that concerns nevertheless exist. These should be explained in training courses and, if necessary, presented transparently in the software. Support from ECHR experts on call during the initial implementation phase could also help to reduce fears.

Second, costs for the implementation are a general challenge. There are no named resources for implementation, as project funding ends with developing the beta version. Now, the implementation is on the side of the interested parties and the software company. Gesulga et al. [8] found costs to be an often-mentioned hurdle to implementation. Costs incurred here would be the software license costs, which according to the software developer would probably amount to 15 Euros per user per month. In addition, there may be costs for hardware, system installation, user training, software maintenance, user support, operating costs for the application server and implementation costs for specific interoperability as well as renewed requirements analysis and adjustments [21].

This is closely related to the third challenge—the planning of the implementation process. It is irritating that project funding exists that focuses on technology development but does not ensure its implementation. The problem of not considering the implementation of research results is also reported from other areas [22]. Andreassen et al. (2015) describe the critical design of projects that always promotes separation between routines in everyday care and the project itself. Thus, the further development of technologies is slowed down, and projects remain an end in themselves [23]. This is also a challenge concerning user participation. While such research projects are intended to support the economy and are certainly designed to be carried out by local companies, this is critical from a research ethics perspective. Users and researchers, as well as developers, invest much time, energy, and commitment in such projects. If the implementation is not guaranteed, one of the greatest dangers in technology development remains unconsidered. Therefore, the implementation should be mandatory for funding approval in future projects. For ECHRs, it makes sense to plan the implementation in a structured way. Different theories, models, and frameworks can be considered regarding implementation. The process of translating research into practice can be supported by process models [24]. One of them is the practical, robust implementation and sustainability model (PRISM). It focuses on four domains (intervention design, recipients, external environment, and implementation and sustainability infrastructure) to plan the implementation [25].

Fourth, it is challenging to support the usage of all PPC professionals of the ECHR. Looking at the statements, it is clear that the ECHR will be used differently for different patients and users. However, even those who only add little information to the ECHR need to be encouraged to do so, as each piece of information can be critical to understanding the complexities of treatment. Compiling all of the information is helpful in PPC due to the varying conditions. In this way, something can be documented in periods of low symptoms, which is critical during a more intensive phase of treatment and must be viewed in the ECHR.

Additionally, it became clear that the ECHR could save time but initially means effort, as information has to be entered manually and, in some cases, documented in addition to the company’s own information system. Increased workload and lack of time are also challenges in implementing electronic health records in other studies [26]. It seems necessary to focus on the advantages and promote the ECHR’s use to set incentives. The lack of planning of the implementation is a frequent obstacle [8]. A particularly significant challenge is that the use of ECHR will not be mandatory. Whereas in hospitals, when an electronic health record is introduced, this is the only possibility for documentation, in the PPC setting described above, the challenge is that all PPC professionals can also document in their primary system. Physicians in medical offices, for example, are already extensively involved in documenting in their system. Additional documentation of critical information and transfer of findings to the ECHR is not mandatory for them. Therefore, seamless and uncomplicated semantic interoperability between such systems and ECHRs is necessary.

As also described in the review by Gesulga et al., a major challenge in implementation is administrative and policy support [8]. Sadly, it is not mandatory in Germany that data on treatment is shared between the different professionals in a common health record over a longer period and are available to all. This is irritating since various studies suggest that sharing information contributes to an increased level of information of the treating persons [27,28]. Information sharing using shared health records reduces duplicate diagnosis [29] and improves multidisciplinary team management of complex patients [30]. Shared medication lists can increase patient safety by offering the actual medication list and frequently detecting drug-drug interactions [31,32]. Therefore, such technology as the ECHR must be used or made more attractive through rewards (compensation).

## 5. Limitations

This study is a first step toward the implementation of an ECHR for PPC in Germany. The study was conducted exclusively in Germany in a small region with a small sample of PPC professionals. This is justified by the highly specialized and not very common setting. In addition, only physicians, nurses, and psychosocial staff from the PPCU, the SOPPC team, and MO were interviewed. It would not have been possible to expand the survey within the project timeframe. However, many other professions (e.g., physical therapy) are involved in the holistic care of patients in the PPC, working in different sectors, and may also need access to the ECHR.

The results cannot be considered representative of the different settings and regionally varied requirements of PPC. Further research with different professions in different regions is needed to specify the need for ECHR in PPC. In addition, the perspective of patients and their relatives should be obtained.

Furthermore, analysis and definition of a PPC core data set for continuity of PPC should be considered.

Pending, moreover, is the planning of implementation. For now, this manuscript provides only an analysis of the weaknesses and strengths of the current state for potential implementation. Detailed planning of how to proceed is needed. In addition, the data analyzed did not explicitly ask about implementation. Rather, this was a topic in the margins of the interviews, and the interview guides brought up many interfaces. However, this resulted in less information being collected in some areas.

## 6. Conclusions

The challenges in terms of implementation are training future users, costs, planning the implementation, and getting all PPC professionals to work with the system to make it useful for everyone. The next step should be to create an implementation plan (using the PRISM model). Critically, the problems are mainly caused by the fact that politically no continuous use of such systems is mandatory and financed, and project funding only ensures development but not implementation and long-term use. A significant change in political circumstances is necessary here to support both practice-oriented research and translation and continuous use.

However, addressing these challenges enables the introduction of a platform that significantly improves PPC by following a coordinated multidisciplinary approach. The ECHR allows for an efficient form of clinical information transmission that contributes to the safety of care and relief of patients and their relatives, as well as all healthcare professionals involved.

## Data Availability

The datasets during and/or analyzed during the current study available from the corresponding author on reasonable request.

## Abbreviations

DT	Design Thinking
ECHR	Electronic cross-facility health record
MC	Main category
MO	Medical office
PPC	Pediatric palliative care
PPCU	Pediatric palliative care unit
SC	Subcategory
SOPPC	Specialized outpatient pediatric palliative care
TA	Think-aloud session

## Appendix A

**Table A1:** Facilitators of and barriers to implementation of a shared electronic cross-facility health record based on CFIR [18].

Adapted CFIR-Construct	Facilitators	Barriers
**I. Intervention Characteristics**		
**Intervention Source** Perception of users about whether the ECHR is ex- or internally developed.	- feeling positive about being involved in the process	- difficult to give critical feedback
**Evidence Strength & Quality** Users’ perceptions of the quality and validity of evidence support the belief that the ECHR will have desired outcomes.	- structured treatment documentation - minimized effort through automatic transfer - improves the safety of care - reduces redundancy - allows for a better overview of the patient - transfer of information in compliance with legal data protection regulations	
**Relative Advantage** Users’ perception of the advantage of implementing the ECHR versus an alternative solution.	- current information sharing is problematic (lack of information, confusion about who is the right contact, lack of information overview, duplicate diagnostics, data overload) - complexity is appropriate - clearly designed - cross-system - designed to extend beyond individual stays	- difficult changeover from analog to digital - fear of errors due to too many users of one ECHR
**Trialability** The ability to test the ECHR on a small scale in the organization and to be able to reverse course (undo implementation) if warranted.	- TA perceived as a workshop for ECHR	
**Complexity** Perceived difficulty of the ECHR, reflected by duration, scope, radicalness, disruptiveness, centrality, and intricacy, and the number of steps required to implement.		- necessity of introduction and further training - necessity to obtain the parents’ consent to use the system and transmit data - costs - high effort - need for someone to control the ECHR
**Design Quality & Packaging** Perceived excellence in how the ECHR is bundled, presented, and assembled.	- intuitive use	- fear of data overload
**Cost** Costs of the ECHR and costs associated with implementing the ECHR include investment, supply, and opportunity costs.		- costs might not be covered
**II. Outer Setting**		
**Patient Needs & Resources** The extent to which the needs of patients and their relatives, as well as barriers and facilitators, to meet those needs are accurately known and prioritized by the organization.	- availability of all information to all parties - eliminating frequent medical history calls → minimized burden on parents - work together on content across sectors	
**Cosmopolitanism** The degree to which the organization is networked with other external organizations.	- regular exchange with 22 different institutions, individuals, and groups - overview of the respective responsible persons per patient in the ECHR was rated positively	
**Peer Pressure** Mimetic or competitive pressure to implement an ECHR, typically due to the fact that most other key peer or competing organizations have already implemented or are in a bid for a competitive edge.	- Competition can lead to the downfall of all systems - ECHR has been available for a long time	- there is already an ECHR in place, which is not complex enough for PPC
**External Policy & Incentives** A broad construct includes external strategies for disseminating the ECHR, including policies and regulations (governmental or other central entity), external mandates, recommendations and guidelines, performance-based pay, collaboratives, and public or benchmark reporting.	- data protection regulations and data security are crucial provisions—current data transfer is not always data protection compliant → advantage of the new system.	- uncertainty whether data-exchange complies with data protection requirements
**III. Inner Setting**		
**Networks & Communications** The nature and quality of social networks and the nature and quality of (in-)formal communications within an organization.	- mails, letters, fax, telephone, and face-to-face meetings - lack of contact persons, unclear responsibilities, and limited accessibility - reasons: referral to specialists, clarification of issues, admission to a facility, and more.	
**Culture** Norms, values, and basic assumptions of the particular organization.		- personal contact is irreplaceable
**Implementation Climate** The receptivity to change, shared receptivity of involved individuals to an intervention, and the extent to which the use of the ECHR will be rewarded, supported, and expected within the organization.		- affinity for technology is very diverse
**Tension for Change** The degree to which users find the current situation intolerable or in need of change.	- current situation is time-consuming and unsatisfactory	
**Compatibility** The degree of tangible fit between the meaning and values attached to the intervention by users, how those align with users’ own norms, values, and perceived risks and needs, and how the intervention fits into existing work processes and systems.		- use of the ECHR is not equally intensive for all individuals - some components might be used selectively, especially by family doctors/pediatrists in MO - large amounts of data from in-/outpatient documentation
**Relative Priority** Users’ shared perception of the importance of the implementation within the organization.	- need for everyone to work with the system	
**Organizational Incentives & Rewards** Extrinsic attractions such as target agreement bonuses, performance appraisals, promotions, and salary increases as well as less tangible attractions such as higher reputation or respect.	- better overview of patients and their disease (history) with ECHR	
**IV. Characteristics of Individuals**		
**Knowledge & Beliefs about the Intervention** Users’ attitudes toward and value of the ECHR as well as familiarity with facts, truths, and principles related to the ECHR.	- TA is helpful to get an impression of the system	
**Self-efficacy** Users’ individual belief in their own capabilities to take action to achieve implementation goals.		- time is scarce in everyone’s daily work; ECHR can save time but involves effort (depending on technology affinity) - assessment of content in TA is sometimes difficult
**Individual Stage of Change** Characterization of the phase a person is in, as he or she uses the intervention skillfully, enthusiastic, and sustainably.	- ECHR is intuitive to use, not complicated	- better overview in paper documentation; one has to get used to the system
**Other Personal Attributes** A broad construct includes other personal characteristics such as ambiguity tolerance, intellectual ability, motivation, values, competence, capacity, and learning style.		- older individuals might be less likely to accept ECHR than younger individuals - individuals from other settings suggested that the system was too complex and time-consuming for family doctors to use

Abbreviations: DT, design thinking; ECHR, electronic cross-sectional health record; PPCU, pediatric palliative care unit; PPC, pediatric palliative care; SOPPC, specialized outpatient pediatric palliative care; TA, think-aloud session.
